# Deformation of a micro-torque swimmer

**DOI:** 10.1098/rspa.2015.0604

**Published:** 2016-01

**Authors:** Takuji Ishikawa, Tomoyuki Tanaka, Yohsuke Imai, Toshihiro Omori, Daiki Matsunaga

**Affiliations:** Department of Bioengineering and Robotics, Tohoku University, 6-6-01, Aoba, Sendai 980-8579, Japan

**Keywords:** ciliate, locomotion, fluid–solid interaction, Stokes flow

## Abstract

The membrane tension of some kinds of ciliates has been suggested to regulate upward and downward swimming velocities under gravity. Despite its biological importance, deformation and membrane tension of a ciliate have not been clarified fully. In this study, we numerically investigated the deformation of a ciliate swimming freely in a fluid otherwise at rest. The cell body was modelled as a capsule with a hyperelastic membrane enclosing a Newtonian fluid. Thrust forces due to the ciliary beat were modelled as torques distributed above the cell body. The effects of membrane elasticity, the aspect ratio of the cell's reference shape, and the density difference between the cell and the surrounding fluid were investigated. The results showed that the cell deformed like a heart shape, when the capillary number was sufficiently large. Under the influence of gravity, the membrane tension at the anterior end decreased in the upward swimming while it increased in the downward swimming. Moreover, gravity-induced deformation caused the cells to move gravitationally downwards or upwards, which resulted in a positive or negative geotaxis-like behaviour with a physical origin. These results are important in understanding the physiology of a ciliate's biological responses to mechanical stimuli.

## 1. Introduction

Ciliates are a group of protozoans characterized by the presence of a large number of short hair-like organelles called cilia. Some ciliates, such as *Paramecium*, show biological reactions when the cell membrane is agitated mechanically [1]. When *Paramecium* bumps against a solid wall with its anterior end, the cell instantaneously inverts the ciliary beat and swims backward for a moment, gyrates about its posterior end, and then returns to its normal forward locomotion. This reaction is called an avoiding reaction. When the posterior end of *Paramecium* is agitated mechanically, however, the cell accelerates its swimming speed for a while; this is called an escape reaction. The frequency and directional responses of the cilia are regulated by membrane potential charges, and the potential charges change when ion channels in the membrane open due to the mechanical stimulus [2]. In the case of *Paramecium*, calcium ion channels are locally distributed around the anterior end, whereas potassium ion channels are locally distributed around the posterior end [3]. The locality of ion channels enables a cell to sense and distinguish mechanical stimuli applied to the membrane at both ends [1].

*Paramecium* is slightly denser than water. It sediments at a speed of about one body length per second in the absence of any ciliary motion. When *Paramecium* swims horizontally, the swimming speed is about 10 body lengths per second. These observations suggest that the swimming velocity in the vertically upward direction may become about nine body lengths per second, whereas that in the vertically downward direction may become about 11 body lengths per second due to the sedimentation effect. Some researchers have actually measured the upward and downward swimming velocities of *Paramecium* [4–6]. Surprisingly, the difference between the upward and downward swimming velocities was much less than the expected value, and the sedimentation effect was considerably reduced. These researchers suggested that a stronger or weaker membrane tension might be induced by a gravity effect, which modified opening or closing of ion channels on the membrane, resulting in a gravitational response in the swimming speed. Similar gravitational responses in swimming speed have been reported for other ciliates, such as *Stylonychia* [7] and *Bursaria* [8]. However, none of these former studies actually measured the membrane tension under gravity. Thus, to understand the physiology of swimming ciliates, the membrane mechanics have to be clarified.

As a mathematical model of a swimming ciliate, a squirmer model has been used by many researchers; it was first proposed by Lighthill [9], and then extended by Blake [10] and Felderhof & Jones [11]. Stone & Samuel [12] provided a simple way of calculating the swimming speed of a squirmer. The surface of the squirmer model represents an envelope of ciliary tips. The translational movement and stretch of the envelope is expressed by time-dependent radial and tangential velocities at the surface of the squirmer. The squirmer model has been used to investigate nutrient uptake properties [13–16], two-body hydrodynamic interactions [17–19], the effect of density stratification [20], collective motions [21–23] and suspension properties [24,25]. These various applications illustrate the utility of the squirmer model in the field of fluid mechanics. However, the original squirmer model is unable to describe the membrane tension of a ciliate, because the squirmer model expresses the velocities at the envelope of ciliary tips, and the cell membrane inside the envelope is excluded from the modelling. Thus, modification of the squirmer model is required to describe both the membrane mechanics and the flow generated by the ciliary beat.

Another type of self-propulsion can be achieved by the deformation of cell shape, as in amoeboid swimming [26]. Many researchers have reported self-propulsion with shape deformations by generating a travelling wave of a constricted region from the cell's anterior end to the posterior end [27–35]. However, most of those studies gave shape deformation as a boundary condition, and the solid mechanics of the cell membrane were again excluded from the modelling.

In this study, we propose a deformable torque swimmer model as a model ciliate. The cell body of a ciliate was modelled as a capsule with a hyperelastic membrane enclosing a Newtonian fluid. Thrust forces due to the ciliary beat were modelled as torques distributed above the cell body. This modelling enabled us to describe both the membrane mechanics and the flow generated by the ciliary beat. We investigated the deformation of a torque swimmer in a Stokes flow regime in a fluid otherwise at rest. The modelling and basic equations are explained in §2. In §3, the effects of the membrane elasticity, the aspect ratio of the cell's reference shape and the density difference between the cell and the surrounding fluid on the swimming behaviours and the membrane tension are investigated. In §4, we conclude our study.

## 2. Basic equations and numerical methods

### (a) A deformable torque swimmer

Modelling a ciliate is a challenging task considering their diversity and complexity [36]. The variety of shapes, both inter- and intra-species, is vast, and cells alter their behaviour depending on various stimuli. Any model capable of being analysed will need to make many simplifications. Here, we propose the simplest model we could think of that both swims and deforms so that fluid and solid mechanics of the ciliate can be analysed non-trivially.

**Figure 1.**
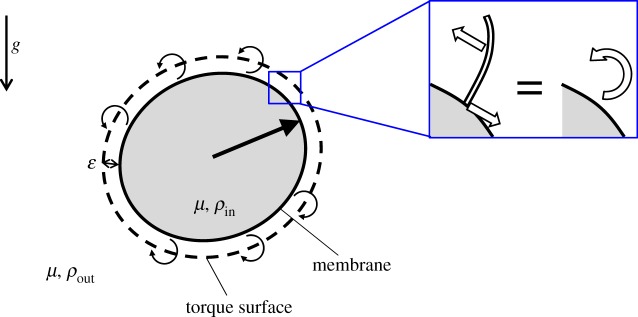
Schematic diagram of a torque swimmer and the problem settings. The torque swimmer consists of a membrane, enclosing fluid with viscosity *μ* and density *ρ*_in_, and a torque surface expressed by the dashed line. The viscosity and density of the external fluid are *μ* and *ρ*_out_, respectively. The bold straight arrow in the cell indicates the swimming direction, and the curved arrows on the torque surface indicate torques. Gravitational direction, *g*, is also shown by an arrow. The inset illustrates the modelling of a thrust force by a torque. (Online version in colour.)

The cell body of a ciliate was modelled as a capsule with a hyperelastic membrane enclosing a Newtonian fluid (figure 1). Because the thickness of the cell membrane is small compared with the cell size and curvature radius, the membrane can be modelled as a two-dimensional hyperelastic surface with surface shear elastic modulus *G*_s_ and area dilation modulus *K*_s_, devoid of bending resistance. The viscosity of the cell membrane was ignored in this study, because steady-state deformation of the membrane was discussed. The viscosity and density of the internal fluid of the membrane are *μ* and *ρ*_in_, respectively, although the internal viscosity does not generate any stress when the cell shape is in the steady state. The external fluid has the same viscosity *μ*, but a different density *ρ*_out_. The density difference between the inside and outside was explicitly considered to investigate the gravity effect on the deformation.

The beat pattern of an individual cilium typically consists of an effective stroke and a recovery stroke [36]. The effective stroke generates a large positive thrust force by straightening the cilium shape during the forward movement, whereas the recovery stroke generates a small negative thrust force by bending the cilium during the backward movement. The asymmetry in the ciliary beat pattern results in a net thrust force to make the cell locomote in a Stokes flow. When a cilium moves in fluid, a viscous traction force is generated on the cilium surface. The surface integral of the traction force is the total drag force induced from the surrounding fluid to the cilium. The total drag force is transmitted to the cell membrane, where the cilium is anchored, and contributes to the membrane deformation. We note that an actual cilium is covered by a membrane, but we neglected such a complex biological structure for simplicity. In the fluid phase, the reaction force of the viscous traction force is induced to the surrounding fluid. The total reaction force acting on the surrounding fluid and the total drag force transmitted to the membrane act in opposite directions with the same magnitude, as shown in the inset of figure 1. The set of two forces may be modelled as a torque generated in the fluid phase slightly above the cell membrane, which is correct in a Stokes flow regime when the elastic force generated on the membrane balances with the viscous traction force, as given by equation (2.9).

The torque distribution can be a function of time and space to mimic the unsteady ciliary beat and the metachronal wave. In this study, however, we used the simplest torque distribution as a first step. We assumed that the torques were exerted at a distance *ε* above the membrane, regardless of the membrane deformation; referred to as a torque surface. The length scale of *ε* can be regarded as about half the length of the cilia. The strength of torque per unit reference area was assumed to be homogeneous and time-invariant. The unsteadiness of the ciliary beat and the inhomogeneity in cilia distribution were ignored. The beat direction was assumed to be in the meridian direction in the reference shape, from the anterior end to the posterior end; thus, the torque vectors were oriented in the latitude direction.

When the membrane was deformed, the strengths and the directions of the torques were changed as follows. Let ***x*** be a material point at the membrane with the outward normal unit vector ***n***(***x***). The corresponding point at the torque surface ***x***′ is given by ***x***′=***x***+*ε****n***(***x***). The torque strength per unit area at ***x***′ was determined by *L*(***x***′)=*L*_0_(***x***′)(Δ*A*_t,0_/Δ*A*_t_|_*x*′_), where *L*_0_ is the torque strength per unit area of the reference shape, and the last fraction indicates the areal ratio of the reference to the deformed torque surface at ***x***′. This assumption means that the local torque strength decreases/increases when the membrane is stretched/compressed. In other words, the number density of cilia is homogeneous at the reference shape, but decreases/increases when the membrane is stretched/compressed. The total amount of torque, given by $\int |\text{L}|\;dS_{t}$, where *S*_t_ denotes the torque surface, was kept constant regardless of the deformation because the number of cilia does not change with the deformation. The direction of torque at the reference shape was in the latitude direction. We assumed that the torque direction in the local coordinate system along the material points was invariant, regardless of the deformation. This assumption means that the beat direction of a cilium relative to the base membrane does not change with the deformation.

Both a spherical cell shape and a prolate ellipsoidal shape were examined in this study. The aspect ratio *α* of the prolate ellipsoid is defined as *α*=*a*_*l*_/*a*_s_, where *a*_*l*_ and *a*_s_ are major and minor axes of the ellipsoid, respectively. The volume of the cell was kept constant regardless of the aspect ratio, i.e. $a^{3}=a_{l}a_{s}^{2}$, where *a* is the radius of the sphere. The torque strength per unit area *L*_0_ was also modified so as to satisfy the same total torque regardless of the aspect ratio. Thus, *L*_0_ of an ellipsoid $L_{0}^{\mathrm{elp}}$ was modified as $L_{0}^{\mathrm{elp}}=L_{0}^{\mathrm{sph}}A_{t}^{\mathrm{sph}}/A_{t}^{\mathrm{elp}}$, where $L_{0}^{\mathrm{sph}}$ is the torque strength per unit area of the sphere, and $A_{t}^{\mathrm{sph}}$ and $A_{t}^{\mathrm{elp}}$ are the torque surface area of the sphere and the ellipsoid, respectively. The same total torque condition may mean that cells with different aspect ratios have the same number of cilia.

The main advantage of the proposed model is its simplicity, which allows us to analyse the motion and deformation of a cell rigorously by using fluid and solid mechanics. The simplicity, however, has some drawbacks in discussing the membrane tension of actual ciliates. A cilium consists of a microtubule-based cytoskeleton, called the axoneme, covered by the plasma membrane. The local membrane tension is thus also affected by the instantaneous shape and motion of the axoneme. In the present modelling, we neglected the detailed shape and the unsteady motion of the axoneme, and smoothed out these effects in time and space. If one wants to quantitatively compare the membrane tension with the experiments, a more detailed modelling might be necessary.

In the following, we explain the basic equations for the deformable torque swimmer. We also provide basic equations for a non-deformable, i.e. rigid, torque swimmer in appendix A for completeness.

### (b) Fluid mechanics

Owing to the small size of typical ciliates, we neglected the inertial effect on the internal and external flow fields as well as the membrane deformation. The flow field is thus governed by the Stokes equation. The velocity ***v*** is given by the integral equation over the deformed membrane *S*_m_ and the torque surface *S*_t_ as [37,38]

2.1 $$\begin{array}{rl} \text{v}(\text{x}) & =-\frac{1}{8\pi \mu }\int_{S_{m}}\text{J}(\text{x},\text{y})\cdot \text{q}(\text{y})\;dS_{m}(\text{y})\\ & \;-\frac{1}{8\pi \mu }\int_{S_{m}}\text{J}(\text{x},\text{y})\cdot (\rho_{\mathrm{in}}-\rho_{\mathrm{out}})gh(\text{y})\text{n}(\text{y})\;dS_{m}(\text{y})\\ & \;-\frac{1}{8\pi \mu }\int_{S_{t}}\text{R}(\text{x},\text{y})\cdot \text{L}(\text{y})\;dS_{t}(\text{y}), \end{array}$$

where *g* is the gravitational acceleration and *h* is the depth. ***J*** and ***R*** are Green functions, defined as

2.2 $$J_{ij}=\frac{\delta_{ij}}{r}+\frac{r_{i}r_{j}}{r^{3}}\;\mathrm{and}\;R_{ij}=\epsilon_{ijk}\frac{r_{k}}{r^{3}},$$

where ***ϵ*** is the unit alternating isotropic tensor, ***r***=***x***−***y*** and *r*=|***r***|. The dynamic condition requires that the load ***q*** must be equal to the viscous traction jump across the membrane

2.3 $$\text{q}(\text{y})=[\sigma_{\mathrm{out}}(\text{y})-\sigma_{\mathrm{in}}(\text{y})]\cdot \text{n}(\text{y}),$$

where ***σ***_in_ and ***σ***_out_ are the stress tensors of the internal and external fluids, respectively.

### (c) Membrane mechanics

Let ***X*** and ***x***(***X***,*t*) be a material point on the membrane in the reference and deformed states, respectively. Assuming negligible bending stiffness, deformation occurs only in the plane of the membrane. The surface deformation gradient tensor ***F***_s_ is then given by

2.4 $$d\text{x}={\text{F}}_{s}\cdot d\text{X}.$$

Local deformation of the membrane can be expressed by the Green–Lagrange strain tensor

2.5 $$\text{E}=\frac{1}{2}({\text{F}}_{s}^{T}\cdot {\text{F}}_{s}-{\text{I}}_{s}),$$

where ***I***_s_ is the tangential projection operator. Two invariants of the in-plane strain tensor ***E*** are given by

2.6 $$I_{1}=\lambda_{1}^{2}+\lambda_{2}^{2}-2\;\mathrm{and}\;I_{2}=\lambda_{1}^{2}\lambda_{2}^{2}-1=J_{s}^{2}-1,$$

where λ_1_ and λ_2_ are the principal stretch ratios. The Jacobian *J*_s_=λ_1_λ_2_ expresses the ratio of the deformed to the reference surface areas.

Assuming that the membrane is a two-dimensional isotropic hyperelastic material, the elastic stresses in an infinitely thin membrane are replaced by elastic tensions. The Cauchy tension ***τ*** is related to the elastic strain energy per unit area *W*_s_(*I*_1_,*I*_2_) as

2.7 $$\tau =\frac{1}{J_{s}}{\text{F}}_{s}\cdot \frac{\partial W_{s}(I_{1},I_{2})}{\partial \text{E}}\cdot {\text{F}}_{s}^{T}.$$

We assumed that a ciliate membrane showed strain-hardening properties with high resistance to areal change. To express these mechanical properties, we used the two-dimensional constitutive law of Skalak given by [39]

2.8 $$W_{s}=\frac{G_{s}}{4}(I_{1}^{2}+2I_{1}-2I_{2}+CI_{2}^{2}),$$

where *G*_s_ is the shear elastic modulus with the units of N m^−1^. The area dilation modulus *K*_s_ can be described as *K*_s_=*G*_s_(1+2*C*). The value of *C* determines the strength of the areal incompressibility. We used *C*=10 throughout this study (see Walter *et al.* [40]); thus, the area dilation ratio was less than about 20% in the parameter range used in this study.

### (d) Numerical method

We used basically the same numerical method as Walter *et al.* [41] and Omori *et al.* [42]. The Stokes flows of the internal and external fluid were solved using a boundary element method. A computational mesh was generated on the membrane as well as on the torque surface, and the Lagrangian positions of the membrane material points ***x***(***X***,*t*) were tracked over time. The in-plane elastic tensions ***τ*** were obtained from the membrane constitutive law. Neglecting the inertia effects of membrane deformation, the static local equilibrium equation of the membrane is given by

2.9 $$\nabla_{s}\cdot \tau +\text{q}=0,$$

where **∇**_s_ is the surface gradient operator. Based on the virtual work principle, the above equation can be rewritten in the weak form as

2.10 $$\int_{S_{m}}\hat{\text{u}}\cdot \text{q}\;dS_{m}=\int_{S_{m}}\hat{\varepsilon }:\tau \;dS_{m}.$$

Here $\hat{\text{u}}$ and $\hat{\varepsilon }=\frac{1}{2}(\nabla_{s}\hat{\text{u}}+\nabla_{s}{\hat{\text{u}}}^{T})$ are the virtual displacement and strain, respectively. A finite-element method was used to solve equation (2.10), and the viscous load ***q*** for a given membrane deformation was derived. Substituting ***q*** into equation (2.1) resulted in a new velocity field ***v***. Once the velocity field ***v*** was computed, the membrane material point ***x*** was updated by means of the kinematic condition ∂***x***/∂*t*=***v***(***x***,*t*) using a second-order Runge–Kutta method, which guaranteed continuity between the membrane velocity and the interfacial fluid velocity.

The membrane was discretized by 32 768 triangular elements with 16 386 nodes. The effect of the mesh resolution is explicitly discussed in appendix B. When the reference membrane shape was an ellipsoid, the computational mesh first generated on a spherical surface was compressed towards the major axis. A computational mesh of the torque surface was then generated from the membrane mesh using the equation ***x***′=***x***+*ε****n***(***x***), where *ε* is set as 0.1*a* throughout the study.

Although equation (2.1) rigorously satisfies the divergence-free conditions of the flow field, numerical error accumulated over a long time duration sometimes causes slight changes in the volume of the internal fluid. To overcome this, we followed Freund [43] and kept a constant internal volume by iteratively modifying the node positions at each time step by ***x***^*n*+1^=***x***^*n*^−3*β*(*V* −*V*_0_)***n***(***x***)/*A*, where *n* is the number of iterations at each time step, *V* is the volume of the capsule, *V*_0_ is the volume of the reference shape and *A* is the surface area of the membrane. Dimensionless parameter *β* controls the convergence, which was set as 0.25 in this study. The volume change of a capsule was kept smaller than 10^−5^ throughout the study.

Let *U*_0_ be the swimming velocity of a spherical torque swimmer, with no deformation, with the torque strength of *L*_0_. We take *U*_0_ as the characteristic velocity of the problem, radius *a* as the characteristic length and *μ*, *G*_s_ and (*ρ*_in_−*ρ*_out_)*g* as characteristic quantities. There are two important dimensionless numbers aside from the aspect ratio. Capillary number (Ca) is defined as Ca=*μU*_0_/*G*_s_, which represents the ratio of viscous to elastic force. The Ca of an actual ciliate is difficult to estimate, because the cell structure is not as simple as a capsule. Here, however, we try to roughly estimate it by using the available data in the literature. The shear elastic modulus *G*_s_ can be correlated to Young's modulus *E* as *G*_s_=*Eh*, where *h* is the membrane thickness. Moreover, in micropipette aspiration experiments, the suction pressure Δ*P* may be expressed as Δ*P*=2*πE*ℓ*ϕ*/(3*a*_p_) (see [44]), where ℓ is the aspiration length, *a*_p_ is the pipette radius and *ϕ* is a coefficient related to the pipette shape. Campillo *et al.* [45] performed micropipette aspiration experiments of *Paramecium*. When they applied a suction pressure of 1 kPa, the aspiration length of the cell cortex was about 10 μm. By inserting these values with *a*_p_=3.5 μm and *ϕ*=2.1, we obtain Young's modulus of about *E*=80 Pa. By assuming the effective thickness of the cell cortex to be in the range 0.1–10 μm, the Ca of *Paramecium*, swimming with a velocity 2 mm s^−1^ in a fluid with viscosity 10^−3^ Pa s, may be derived in the range 0.25–0.0025. In this study, we examined the parameter range of 0≤Ca≤0.5.

Gr is another dimensionless number defined as

2.11 $$\text{Gr}=\frac{(\rho_{\mathrm{in}}-\rho_{\mathrm{out}})ga^{2}}{\mu U_{0}},$$

which represents the ratio of gravity to viscous force. Gr also represents the ratio of sedimentation to swimming speed, which is useful to discuss the upward or downward swimming under gravity. We define Gr_0_ such that the swimming speed and the sedimentation speed of a spherical swimmer are equivalent, i.e.

2.12 $$\frac{4}{3}\pi (\rho_{\mathrm{in}}-\rho_{\mathrm{out}})ga^{3}=6\pi \mu aU_{0},$$

which gives Gr_0_=4.5. We propose Gr/Gr_0_ as the simple measure of the sedimentation effect on swimming. Gr/Gr_0_<1 indicates that the sedimentation velocity is smaller than the swimming velocity, whereas Gr/Gr_0_>1 indicates that the sedimentation velocity is larger than the swimming velocity. The sedimentation velocity of *Paramecium* is about $\frac{1}{10}$ the swimming velocity, so Gr/Gr_0_ of *Paramecium* is about 0.1. In this study, we examined the parameter range of 0≤Gr/Gr_0_≤0.5.

## 3. Results and discussion

### (a) Spherical swimmer without the gravity effect

**Figure 2.**
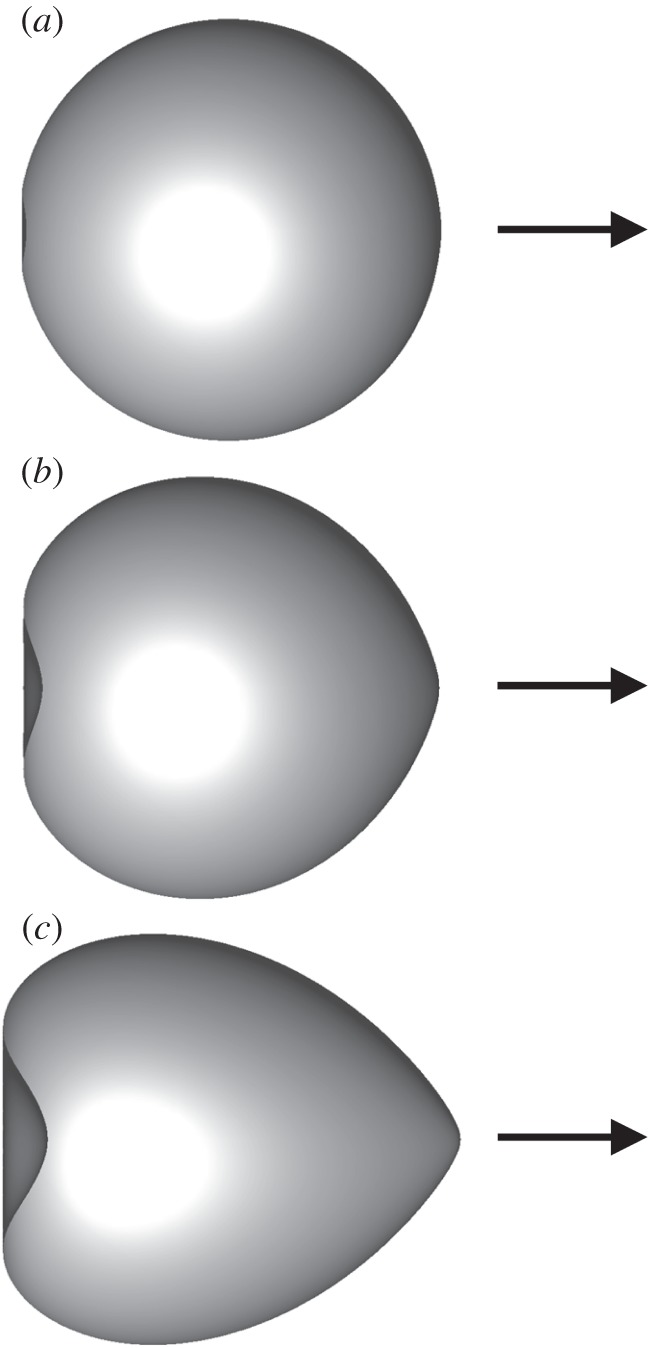
Shape of a torque swimmer with Ca= (*a*) 0.001, (*b*) 0.05 and (*c*) 0.5, when the deformation reached the steady state (*α*=1, Gr=0). Arrows indicate the swimming direction.

First, we calculated the deformation of a spherical cell swimming in a still fluid with the same density, i.e. in the absence of gravity effects. We referred to the swimmer as spherical, given that its reference shape was spherical, although its actual shape changes when Ca>0. Figure 2 shows the shape of a spherical swimmer when Ca=0.001 and 0.5 (Gr=0). When Ca=0.001, the shape is almost spherical, because the viscous force is much smaller than the elastic force of the membrane. We see a little deformation only at the posterior end in this case. When Ca=0.5 (figure 2*b*), however, we see clearly that the cell deformed, like an axisymmetric heart shape; the anterior end was sharpened, whereas the posterior end was dented. The torque distribution at the anterior end generated outward radial velocity, which made the sharpened anterior end. The torque distribution at the posterior end generated an inward radial velocity, which induced buckling of the posterior end.

**Figure 3.**
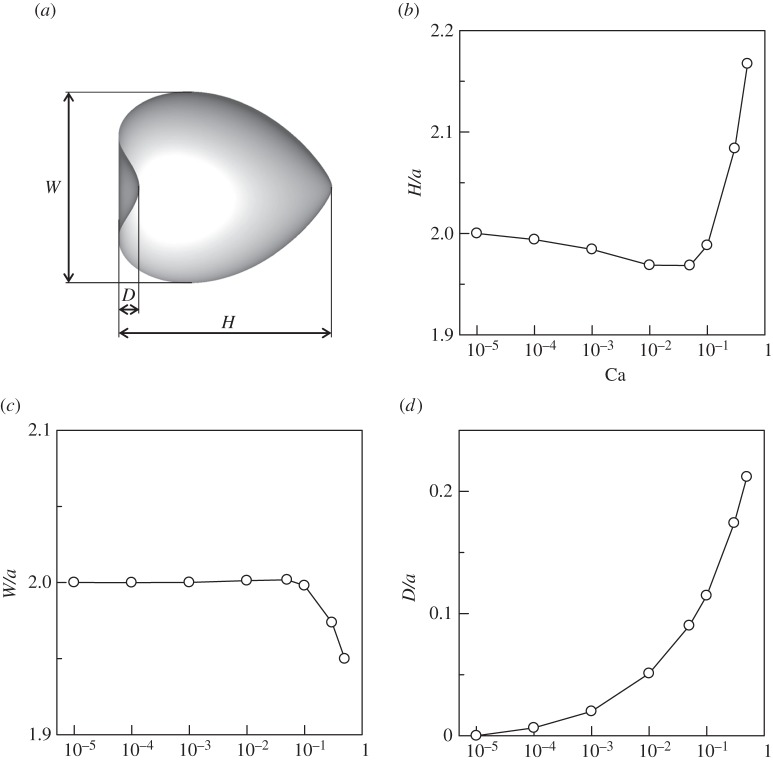
Effect of Ca on the deformation of a torque swimmer (*α*=1, Gr=0). (*a*) Definition of *L*, *W* and *D*, (*b*) length *H*, (*c*) width *W* and (*d*) dent *D*.

Deformation of the spherical cell was evaluated by three parameters; length *H*, width *W* and dent *D* (figure 3). In the small Ca limit, the cell shape converges to the reference shape, which has values of *H*=2, *W*=2 and *D*=0. We see from figure 3*b* that *H* increases rapidly as Ca increased when Ca>0.1. *W*, however, decreased rapidly as Ca increased when Ca>0.1 (figure 3*c*). *D* increased gradually as Ca increased over the entire Ca regime. Cell deformation is strongly dependent on Ca.

**Figure 4.**
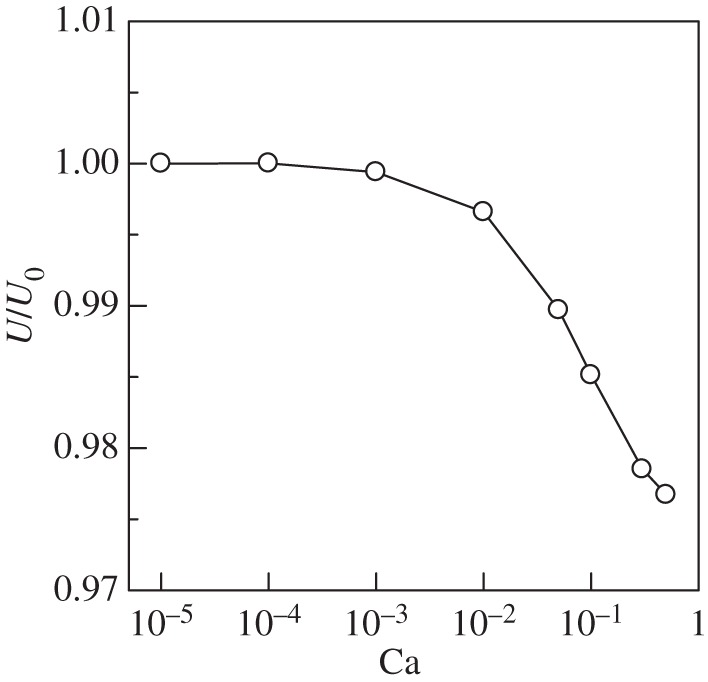
Effect of Ca on the swimming velocity *U*(*α*=1, Gr=0).

When the cell was deformed, the swimming velocity changed (figure 4). We note that the total amount of torque, given by $\int |\text{L}|\;dS_{t}$, was kept constant in all cases (see §2a). We see that the swimming velocity *U* decreased slightly from that of the undeformed one, *U*_0_. When the cell deformed like a heart shape, the torque around the posterior end generated the load ***q*** opposite to the swimming direction. Moreover, additional drag could be generated by the largely deformed cell surface. These effects might slow the swimming speed.

**Figure 5.**
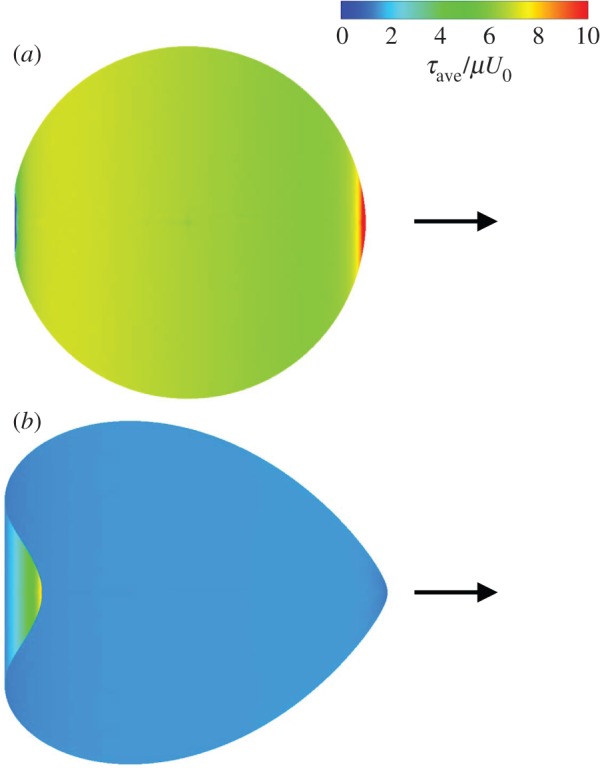
In-plane isotropic tension *τ*_ave_ of the torque swimmer with Ca= (*a*) 0.001 and (*b*) 0.5 (*α*=1, Gr=0). Arrows indicate the swimming direction. (Online version in colour.)

Next, we calculated the membrane tension of the spherical swimmer. We show the results of in-plane isotropic tension defined as *τ*_ave_=(*τ*_1_+*τ*_2_)/2, where *τ*_1_ and *τ*_2_ are the in-plane principal tensions, because the opening/closing of an ion channel at a membrane can be regulated by the isotropic tension acting on the membrane [46]. Figure 5 shows the in-plane isotropic tension *τ*_ave_ with Ca=0.001 and 0.5. The colour contour of *τ*_ave_ was normalized by the viscous force *μU*_0_, and thus increasing Ca corresponds to decreasing the membrane shear elastic modulus *G*_s_. Around the equator of the cell, stronger *τ*_ave_ was generated in the Ca=0.001 case than in the Ca=0.5 case. This is because the membrane with Ca=0.5 deformed so as to follow the fluid flow, and a weaker viscous force was exerted on the membrane. In the Ca=0.001 case, *τ*_ave_ was larger at the anterior end than at the posterior end. In the Ca=0.5 case, on the other hand, *τ*_ave_ was larger at the posterior end than at the anterior end. These results illustrate that the tension was inhomogeneously distributed on the cell membrane and was strongly affected by Ca.

**Figure 6.**
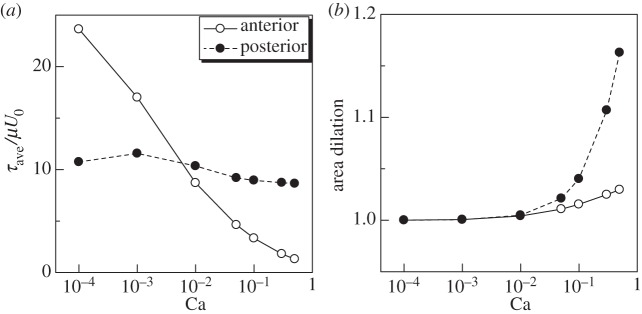
Effect of Ca on the membrane tension and the area dilation ratio of a torque swimmer (*α*=1, Gr=0). The values at the anterior and the posterior ends are plotted. (*a*) In-plane isotropic tension *τ*_ave_ and (*b*) area dilation ratio.

In figure 6*a*, *τ*_ave_ at the anterior and the posterior ends with various Ca conditions are shown. We see that *τ*_ave_ at the anterior end decreased monotonically as Ca increased, whereas *τ*_ave_ at the posterior end was almost constant with changing Ca. Thus, the cell membrane of a ciliate experiences stronger isotropic tension at the posterior end than at the anterior end when Ca > 10^−2^, but the other way around when Ca<10^−3^. We also show the area dilation ratio in figure 6*b*. Basically, the area dilation ratio increased as Ca increased, because the membrane became softer as Ca increased. The area dilation ratio was larger at the posterior end when Ca was large. This is because *τ*_ave_ of the posterior end was larger than that of the anterior end (figure 6*a*).

### (b) Ellipsoidal swimmer without the gravity effect

**Figure 7.**
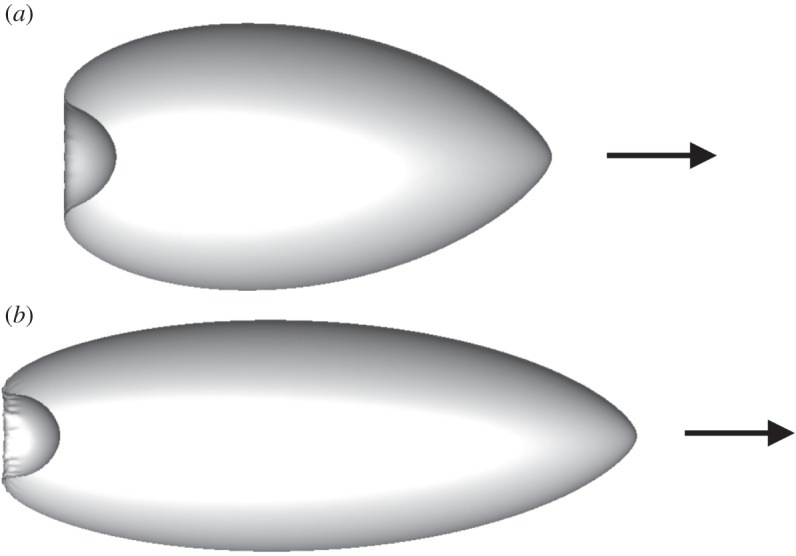
Shape of a torque swimmer with *α*= (*a*) 2 and (*b*) 3, when the deformation reached the steady state (Ca=0.5, Gr=0). Arrows indicate the swimming direction.

In this section, we vary the aspect ratio *α* of the reference cell shape, given that some ciliates, such as *Paramecium* and *Tetrahymena*, are not spherical but rather ellipsoidal. The gravity effect is neglected in this section, i.e. Gr=0. Figure 7 shows the shape of an ellipsoidal swimmer with *α*=2 and 3 (Ca=0.5). We see that the cell deformed, like an axisymmetric heart shape, similar to figure 2*b*.

**Figure 8.**
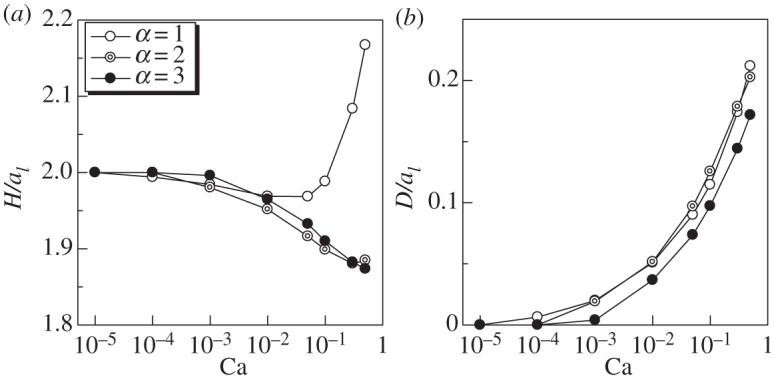
Effect of aspect ratio *α* on the deformation of a torque swimmer (Gr=0). (*a*) Length *H* and (*b*) dent *D*.

The length *H* and the dent *D* of ellipsoidal swimmers are shown in figure 8. *H* of *α*=2 and 3 decreased monotonically as Ca increased, although that of *α*=1 first decreased and then increased as Ca increased. These results illustrate that *α* caused a qualitative difference in the cell deformation. *D* increased as Ca increased regardless of *α*. *D* of *α*=3 was slightly smaller than that of *α*=1 and 2.

**Figure 9.**
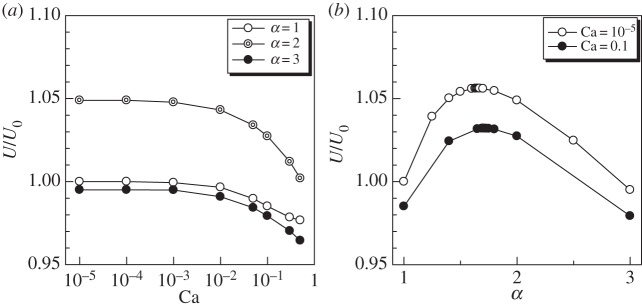
Effect of aspect ratio *α* on the swimming velocity *U*(Gr=0). (*a*) Effect of Ca with *α*=1, 2 and 3 and (*b*) effect of *α* with Ca=0.1 and 10^−5^.

In figure 9, we show the effect of *α* on the swimming velocity *U*. By increasing Ca, *U* decreased monotonically in all *α* conditions (figure 9*a*). The swimming velocity of *α*=2 was larger than that of *α*=1 and 3. We thus calculated the swimming velocity by changing *α*, as shown in figure 9*b*. In changing *α*, the cell volume was kept constant, and the torque strength per unit area was modified so as to satisfy the same total torque condition, i.e. $L_{0}^{\mathrm{elp}}=L_{0}^{\mathrm{sph}}A_{t}^{\mathrm{sph}}/A_{t}^{\mathrm{elp}}$ (see §2a). These conditions may mean that the cells with different *α* have the same volume of cytoplasm and the same number of cilia, but their aspect ratios and surface areas are different. Under these conditions, we found that the cell with *α* of about 1.7 had the maximum swimming velocity. As *α* increased under the same volume condition, the viscous drag coefficient of the particle also increased, which had the effect of slowing down the swimming velocity. The thrust force, however, oriented towards the swimming direction more effectively as *α* increased, which had the effect of speeding up the swimming velocity. These two conflicting effects may be the reason for the peak shown in figure 9*b*. Bourot [47] investigated the rigid body shape of a given volume with the smallest drag in Stokes flow. He found that the optimal body has an aspect ratio of about 2, which is slightly larger than the present results. The deviation may come from the difference in problem settings between the two studies.

**Figure 10.**
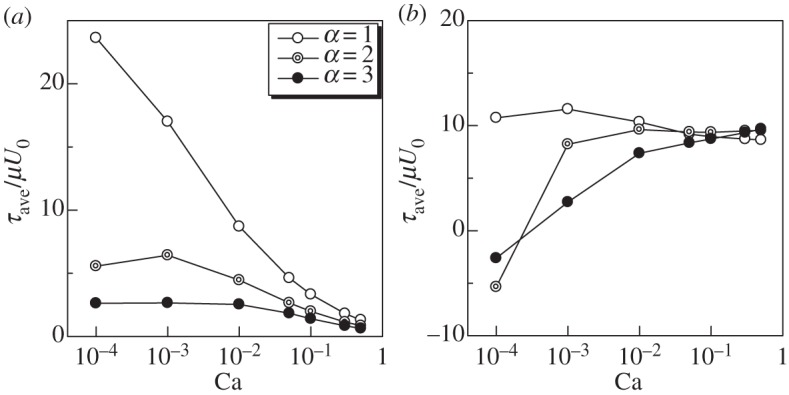
Effect of aspect ratio *α* on the in-plane isotropic tension *τ*_ave_ (Gr=0). (*a*) Anterior and (*b*) posterior ends.

The in-plane isotropic tension *τ*_ave_ with *α*=1, 2 and 3 is shown in figure 10. The value of *τ*_ave_ at the anterior end decreased as *α* increased. *τ*_ave_ at the posterior end, however, showed complex tendencies due to the buckling effect. When *α*=2 and 3, *τ*_ave_ at the posterior end increased as Ca increased. The aspect ratio considerably affected the isotropic tensions.

### (c) Effects of gravity on shape and tension

**Figure 11.**
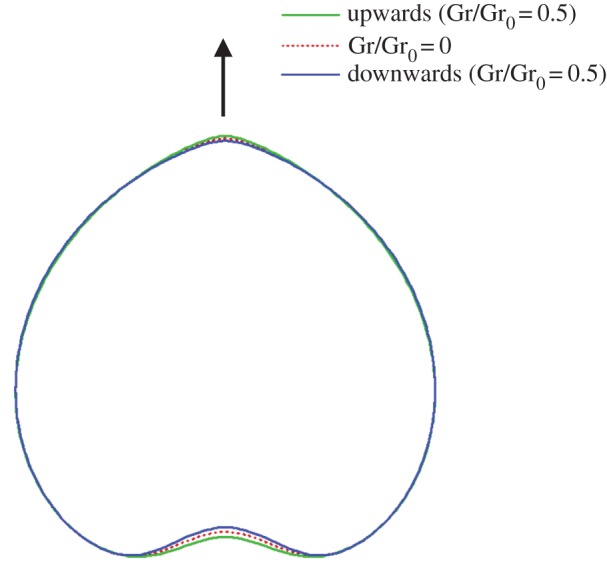
Effect of gravity on the deformation of a torque swimmer with Ca=0.1 (*α*=1). The arrow indicates the swimming direction. (Online version in colour.)

Finally, we investigated the gravity effect caused by the density difference between the internal and external fluids. Vertically upward and downward swimming of a spherical cell with Gr/Gr_0_=0.5 and Ca=0.1 were calculated, and the cell deformation was compared with the Gr=0 case (figure 11). To readily compare the three shapes, the swimming direction, indicated by the arrow, was identical in all cases, i.e. the results of downward swimming were inverted. We see a slight change in the cell shape around the anterior and posterior ends. The anterior end was sharpened more strongly in upward swimming than in downward swimming. The dent at the posterior end became deeper in downward swimming than in upward swimming. These differences in cell shape affected the membrane tension, and eventually may affect the control of opening/closing of ion channels on the cell membrane.

**Figure 12.**
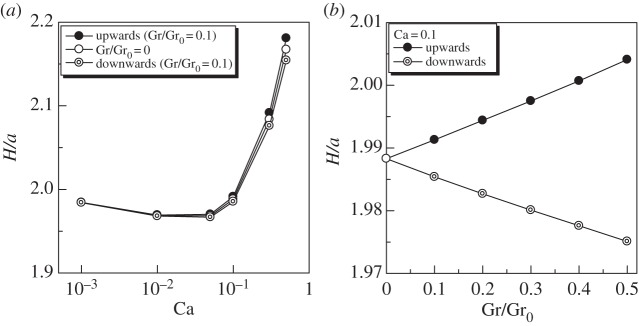
Effect of gravity on the length *H* of a torque swimmer (*α*=1). (*a*) Effect of Ca and (*b*) effect of Gr.

The effect of gravity on the cell length is shown in figure 12 (*α*=1). We see that the upward swimming cell with Gr/Gr_0_=0.1 was elongated slightly more than the neutral cell with Gr=0. The downward swimming cell with Gr/Gr_0_=0.1, however, was compressed slightly more than the neutral cell. The effect of Gr was almost linear in the parameter range Gr ≤ 0.5, as shown in figure 12*b*.

**Figure 13.**
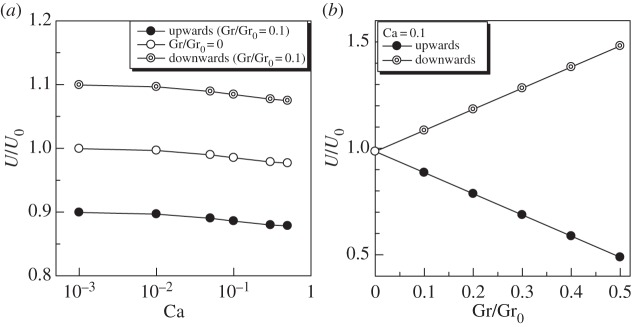
Effect of gravity on the swimming velocity *U* of a torque swimmer (*α*=1). (*a*) Effect of Ca and (*b*) effect of Gr.

The swimming velocity was affected considerably by gravity (figure 13). When Gr/Gr_0_=0.1 (figure 13*a*), *U* increased about 10% in downward swimming, whereas it decreased about 10% in upward swimming. These results indicate that the overall swimming velocity was approximately the superposition of the sedimentation velocity and the swimming velocity without a gravity effect. In reality, however, the difference between the upward and downward swimming velocities of *Paramecium* was much less than the superimposed value [4–6]. The inconsistency of the present results clearly illustrates that some biological responses to the gravity field should be introduced to explain the experimental observations.

**Figure 14.**
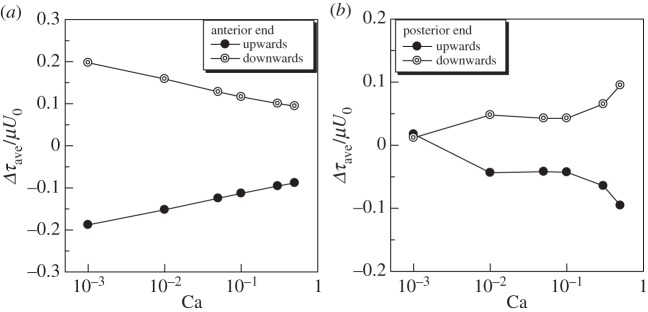
Difference in the isotropic tension between the neutral case (Gr=0) and the upward or downward swimming case with Gr=0.1 (*α*=1). (*a*) Anterior and (*b*) posterior ends.

To discuss the opening/closing of an ion channel at the membrane, we investigated the effect of gravity on the in-plane isotropic tension *τ*_ave_. Here, we focus on the difference in *τ*_ave_ from the Gr=0 case, i.e. Δ*τ*_ave_. We see from figure 14*a* that larger Δ*τ*_ave_ was generated in the downward swimming than in the upward swimming at the anterior end. In the case of *Paramecium*, calcium ion channels are locally distributed around the anterior end. Opening of the calcium ion channels decelerates the swimming speed, and sometimes causes the avoiding reaction. Our results of larger Δ*τ*_ave_ in downward swimming indicate that downward swimming may be decelerated by opening a larger number of calcium ion channels. Smaller Δ*τ*_ave_ in upward swimming, on the other hand, indicates that upward swimming may be accelerated by closing a larger number of calcium ion channels. These results suggest that the difference between upward and downward swimming velocities may be reduced by the opening/closing of calcium ion channels distributed locally around the anterior end. We note, however, that the above discussion is just biomechanical speculation at this stage, and experimental evidence will be needed in future studies.

The effect of gravity on Δ*τ*_ave_ at the posterior end is shown in figure 14*b*. We again see that a larger *τ*_ave_ was generated in downward swimming than in upward swimming. In the case of *Paramecium*, potassium ion channels are locally distributed around the posterior end. Opening of the potassium ion channels accelerates the swimming speed, and sometimes causes the escape reaction. The present results of a larger Δ*τ*_ave_ in downward swimming indicate that downward swimming may be accelerated by opening a larger number of potassium ion channels. A smaller Δ*τ*_ave_ in upward swimming, on the other hand, indicates that upward swimming may be decelerated by opening a smaller number of potassium ion channels. This hypothesis, however, conflicts with the experimental observations. We thus speculate that opening/closing of the potassium ion channels is not sensitive in the Δ*τ*_ave_ range shown in figure 14*b*, and the potassium ion channels do not play a major role in the gravitational response in the swimming speed. We again note that experimental evidence will be needed in future studies to prove the biomechanical speculation.

### (d) Orientation change due to the gravity effect

**Figure 15.**
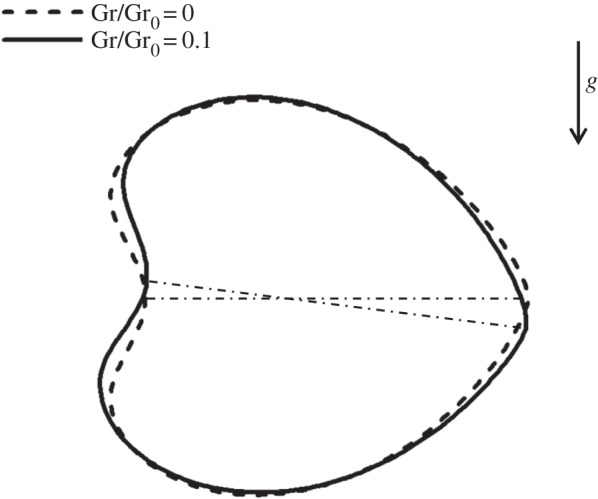
Shape of a torque swimmer, initially oriented horizontally, at *tU*_0_/*a*=450 (Ca=0.3, *α*=1). The arrow indicates the gravitational direction. The cell with Gr/Gr_0_=0 swam horizontally, whereas the cell with Gr/Gr_0_=0.1 was oriented downwards.

Finally, we discuss the orientation change of a spherical swimmer initially directed horizontally under gravity. When there is no gravity effect, the deformation becomes axisymmetric around the cell's orientation vector, and no directional change occurs. When there is a gravity effect, however, the deformation of the horizontally directed cell is no longer axisymmetric, and directional change may occur. Figure 15 shows the shape of a spherical cell, initially directed horizontally, at *tU*_0_/*a*=450. The dashed-dotted line indicates the cell's orientation, which passes through the material points initially at the anterior and posterior poles. We see from figure 15 that the cell with Gr/Gr_0_=0.1 is directed slightly downwards. The orientation change was caused by the asymmetric deformation of the membrane due to the gravity effect.

**Figure 16.**
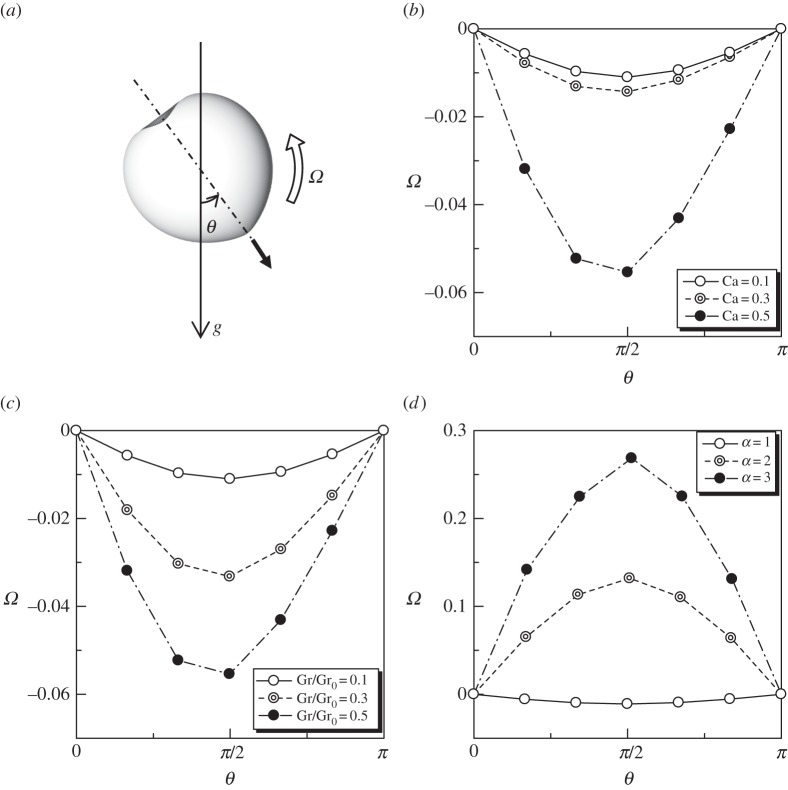
Rotational velocity *Ω* of a torque swimmer with orientation *θ* under various conditions. (*a*) Definition of *θ* and *Ω* relative to the gravitational direction *g*, (*b*) effect of Ca on *Ω* (*α*=1, Gr/Gr_0_=0.1), (*c*) effect of Gr on *Ω* (Ca=0.1, *α*=1) and (*d*) effect of *α* on *Ω* (Ca=0.1, Gr/Gr_0_=0.1).

In order to clarify the gravity effect on the orientation change in more detail, we calculated the rotational velocity *Ω* of a torque swimmer under various conditions. *θ* is the instantaneous angle between the gravity axis and the cell's orientation vector, as shown in figure 16*a*. *Ω* was calculated from the time change of the orientation vector when the cell shape was at the quasi-steady state. *Ω* becomes positive when the cell rotates gravitationally upwards, as shown in figure 16*a*. The effect of Ca on *Ω* is shown in figure 16*b* (*α*=1, Gr/Gr_0_=0.1). We see that *Ω* was small at around *θ*=*π*/2 and negative in the whole *θ* regime. The *Ω* value decreased as Ca was increased. These results indicate that the cell with *α*=1 and Gr/Gr_0_=0.1 was directed gravitationally downwards gradually due to the gravity effect. Such a tendency may be called positive geotaxis, given that cells tend to swim gravitationally downwards, on average.

Figure 16*c* shows the effect of Gr on the rotational velocity *Ω*. We see that *Ω* decreases as Gr was increased and is negative in the whole *θ* regime. In the parameter range used in this study, the spherical torque swimmer always showed positive geotaxis. When the aspect ratio *α* was altered, however, the results changed dramatically, as shown in figure 16*d*. In the case of *α*=2 and 3, *Ω* was positive in the whole *θ* regime. The results indicate that the cell with *α*=2 or 3 was directed gravitationally upwards gradually due to the gravity effect. Such a tendency may be called negative geotaxis, given that cells tend to swim gravitationally upwards, on average. We note that some single-celled organisms are known to use gravity and buoyancy forces for taxis [48,49]. The geotaxis behaviour reported in this study is different from the former studies, though it also has a physical origin. In this study, the positive or negative geotaxis was caused by the deformation of the cell body.

## 4. Conclusion

In this study, we investigated the deformation of a ciliate swimming freely in a fluid otherwise at rest. We proposed a torque swimmer as a model ciliate, where the cell body was assumed as a capsule with a hyperelastic membrane enclosing a Newtonian fluid. Thrust force due to the ciliary beat was modelled as torque distributed above the cell body. The effects of membrane elasticity, the aspect ratio of the cell's reference shape, and the density difference between the cell and the surrounding fluid were investigated. The results showed that the cell deformed, like a heart shape, when Ca was sufficiently large, and the swimming velocity decreased as Ca increased. By changing the aspect ratio while keeping the same total torque and the same cell volume, the swimming velocity became maximal at an aspect ratio of about 1.7. The membrane isotropic tension decreased at the anterior end, whereas it was almost constant at the posterior end, as Ca increased. Under the influence of gravity, the membrane tension at the anterior end decreased in the upward swimming while it increased in the downward swimming. Moreover, gravity-induced deformation tended to direct a cell gravitationally downwards or upwards, resulting in a positive or negative geotaxis-like behaviour with a physical origin. These results are important in understanding the physiology of a ciliate's biological responses to mechanical stimuli.

## Figures and Tables

**Figure 17.**
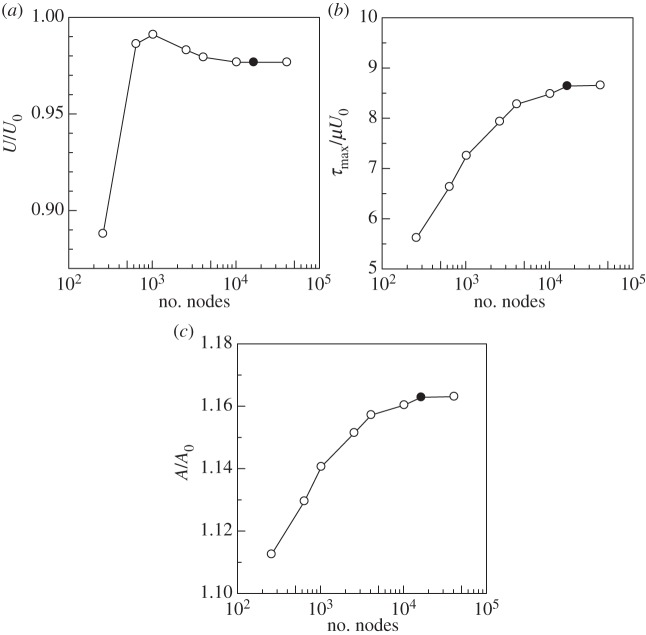
Effects of node number on the deformation and swimming motion of a torque swimmer (*α*=1, Ca=0.5, Gr=0). The black circle indicates the node number of 16 386, which was used throughout the study. (*a*) Swimming velocity *U*, (*b*) maximum tension $\tau_{\max}$ and (*c*) total surface area of the membrane *A*.

## Data accessibility

This work has no accompanying data.

## Appendix A. Basic equations for a non-deformable torque swimmer

In this appendix, we provide basic equations for a non-deformable torque swimmer for completeness of the paper. When the cell body is assumed as rigid, the solid mechanics in §2c can be skipped, and the basic equations are significantly simplified.

The velocity ***v*** is given by the integral equation over the rigid body surface *S*_*r*_ and the torque surface *S*_t_ as

A 1 $$\text{v}(\text{x})=-\frac{1}{8\pi \mu }\int_{S_{r}}\text{J}(\text{x},\text{y})\cdot {\text{f}}_{\mathrm{out}}(\text{y})\;dS_{r}(\text{y})-\frac{1}{8\pi \mu }\int_{S_{t}}\text{R}(\text{x},\text{y})\cdot \text{L}(\text{y})\;dS_{t}(\text{y}),$$

where the traction force ***f***_out_ is defined as ***f***_out_=***σ***_out_(***y***)⋅***n***(***y***). In equation (A1), the effect of gravity is implicitly included through the distribution of ***f***_out_. The velocity has to satisfy the boundary condition given by

A 2 $$\text{v}(\text{x})=\text{U}+\Omega \wedge (\text{x}-{\text{x}}_{0}),\;\text{x}\in S_{r},$$

where ***U*** and ***Ω*** are the translational and rotational velocities of the rigid body, respectively, and ***x***_0_ is the centre of the body.

It is supposed that the rigid body is subjected to known external force ***F***_*r*_ and torque ***T***_*r*_. The equilibrium conditions for the rigid body are

A 3 $${\text{F}}_{r}=\int_{S_{r}}{\text{f}}_{\mathrm{out}}(\text{x})\;dS_{r}\;\mathrm{and}\;{\text{T}}_{r}=\int_{S_{r}}\text{x}\wedge {\text{f}}_{\mathrm{out}}(\text{x})\;dS_{r}.$$

The gravity effect should be included in ***F***_*r*_, when the whole body is not neutrally buoyant. For the numerical methods to solve the above equations, see Ishikawa *et al.* [17].

## Appendix B. Effect of mesh resolution on the numerical results

The aim of this appendix is to clarify the effect of mesh resolution on the numerical results. The total number of nodes on the cell membrane was varied from 258 to 40 962, and the results of swimming velocity *U*, maximum in-plane tension in the whole membrane $\tau_{\max}$, and the total surface area *A* normalized by that of the reference shape *A*_0_ are shown in figure 17. We see that all values converged as the number of nodes increased. Considering computational efficiency, while keeping numerical accuracy, we decided to use 16 386 nodes throughout the study.
